# Novel Approach to Assess Cardiac Function Using Systolic Performance and Myocardial Performance Indices From Simultaneous Electrocardiography and Phonocardiography Recordings in Dogs With Various Stages of Myxomatous Mitral Valve Disease

**DOI:** 10.3389/fvets.2021.741115

**Published:** 2021-10-21

**Authors:** Karlo Romano B. Gicana, Tuchakorn Lertwanakarn, Kittipong Tachampa

**Affiliations:** ^1^The International Graduate Program of Veterinary Science and Technology, Faculty of Veterinary Science, Chulalongkorn University, Bangkok, Thailand; ^2^Department of Veterinary Clinical Sciences, College of Veterinary Medicine, University of the Philippines Los Baños, Los Baños, Philippines; ^3^Department of Physiology, Faculty of Veterinary Medicine, Kasetsart University, Bangkok, Thailand; ^4^Department of Physiology, Faculty of Veterinary Science, Chulalongkorn University, Bangkok, Thailand

**Keywords:** cardiac time intervals, dogs, electrocardiography, MMVD, myocardial performance index, phonocardiography, systolic performance index, Tei index

## Abstract

**Background and Objective:** Myxomatous mitral valve disease (MMVD) progression entails changes in the structural and functional properties of the heart affecting cardiac timings and intervals within the cardiac cycle. Conventionally, echocardiography is used to determine the cardiac time intervals (CTIs) including systolic and myocardial performance indices (SPI and MPI) in evaluating cardiac function. Alternatively, these CTIs can also be measured using simultaneous recordings of electrocardiography (ECG) and phonocardiography (PCG), but their values in different MMVD stages remain to be established. This study aimed to establish and prove the use of derived SPI and MPI from a dedicated device as a novel approach to assess cardiac function in different stages of MMVD dogs.

**Materials and Methods:** A prospective study in 52 dogs with different MMVD stages measured the CTIs using a novel device. These were compared and correlated with standard echocardiographic parameters. The predictive value of SPI and three new proposed formulas to estimate MPI (i.e., F1, F2, and F3) in association with asymptomatic from symptomatic MMVD dogs were investigated.

**Results:** Our findings revealed that CTI parameters measured from a novel device including QS1, QS2, S1S2, MPI-F1, and MPI-F2 were altered at different stages of MMVD. The SPI and all proposed MPI formulas were comparable with the systolic time interval and Tei index from echocardiography. In addition, the SPI, MPI-F1, and MPI-F2 were significantly correlated with the Tei index. However, the SPI was not able to differentiate the various stages of MMVD. Conversely, only the MPI-F1 (i.e., (QS1 + S2)/S1S2) demonstrated good predictive accuracy when compared between asymptomatic and symptomatic MMVD dogs similar to the Tei index. Moreover, this formula was able to differentiate stages B1 and C with remarkable predictive accuracy, higher sensitivity, and high specificity when compared with the Tei index.

**Conclusion:** We have successfully described the CTI parameters in different MMVD stages using simultaneous ECG and PCG recordings in dogs. Furthermore, we have proven that the concept of using the newly proposed parameters from a novel device is equivalent to the Tei index. Thus, we established a novel approach to evaluate cardiac function and its supportive use in the diagnosis of MMVD patients.

## Introduction

The most commonly acquired cardiac disease in dogs is a myxomatous mitral valve disease (MMVD) (1). Mitral valves (MVs) can undergo degenerative pathologic changes due to many factors including genetics (2) and age (3). Aging caused cell-mediated remodeling of MV leaflets through various signaling pathways (3, 4). Changes in the extracellular matrix were brought by alterations in the type of cells and the density across different layers of the MV and other related structures (3–5). Eventually, these lesions affect the structural, mechanical, and hemodynamic functions of the heart (6–8). Disease progression involves heart remodeling affecting systolic (9) and/or diastolic (10, 11) functions. The MMVD is classified into four different stages (A–D) according to the American College of Veterinary Internal Medicine (ACVIM) (12). Stage A refers to dog breeds with a genetic predisposing to MMVD but has no structural abnormality yet. In asymptomatic dogs with murmur heart sound (stage B), it can be further classified as without a cardiac enlargement (substage B1) or with a cardiac enlargement (substage B2). Stage C refers to a group of dogs with signs of congestive heart failure (CHF). When these dogs develop refractoriness to treatment in the presence of advanced heart failure (HF), stage D may apply to this category.

Echocardiography is a gold standard tool to evaluate MV lesion, mitral regurgitation (MR) severity, cardiac remodeling, and hemodynamic impairment associated with MMVD progression (8, 13, 14). It can also provide information on the various properties of the heart associated with its cardiac function through cardiac time interval (CTI) parameters. Changes in CTIs were observed in valvular diseases in both humans (15, 16) and dogs (17, 18). Among CTI parameters, systolic performance index (SPI) and myocardial performance index (MPI) are both simple and reliable parameters derived from pulsed-wave or tissue Doppler echocardiography (19–21). Both parameters have been shown to be clinically useful in approximating left ventricular (LV) function (i.e., the systolic and diastolic performances) in various human and canine cardiac diseases (22, 23). The SPI parameter is defined as the ratio between the pre-ejection period (PEP) and LV ejection time (LVET) (24), whereas MPI is the sum of the isovolumic relaxation time (IVRT) and the isovolumic contraction time (IVCT) divided by LVET. Alternatively, it can be calculated in the echocardiographic machine using the time from MV close to open duration (MCOdur) and the LVET as the following equation: (MCO – LVET)/LVET. MPI has been shown to increase with the progression of MR in dogs (17) and is suggested as a useful parameter to reflect LV dysfunction (25). Nevertheless, MPI in MMVD dogs with various ACVIM stages has not been well-documented.

**Figure 1:**
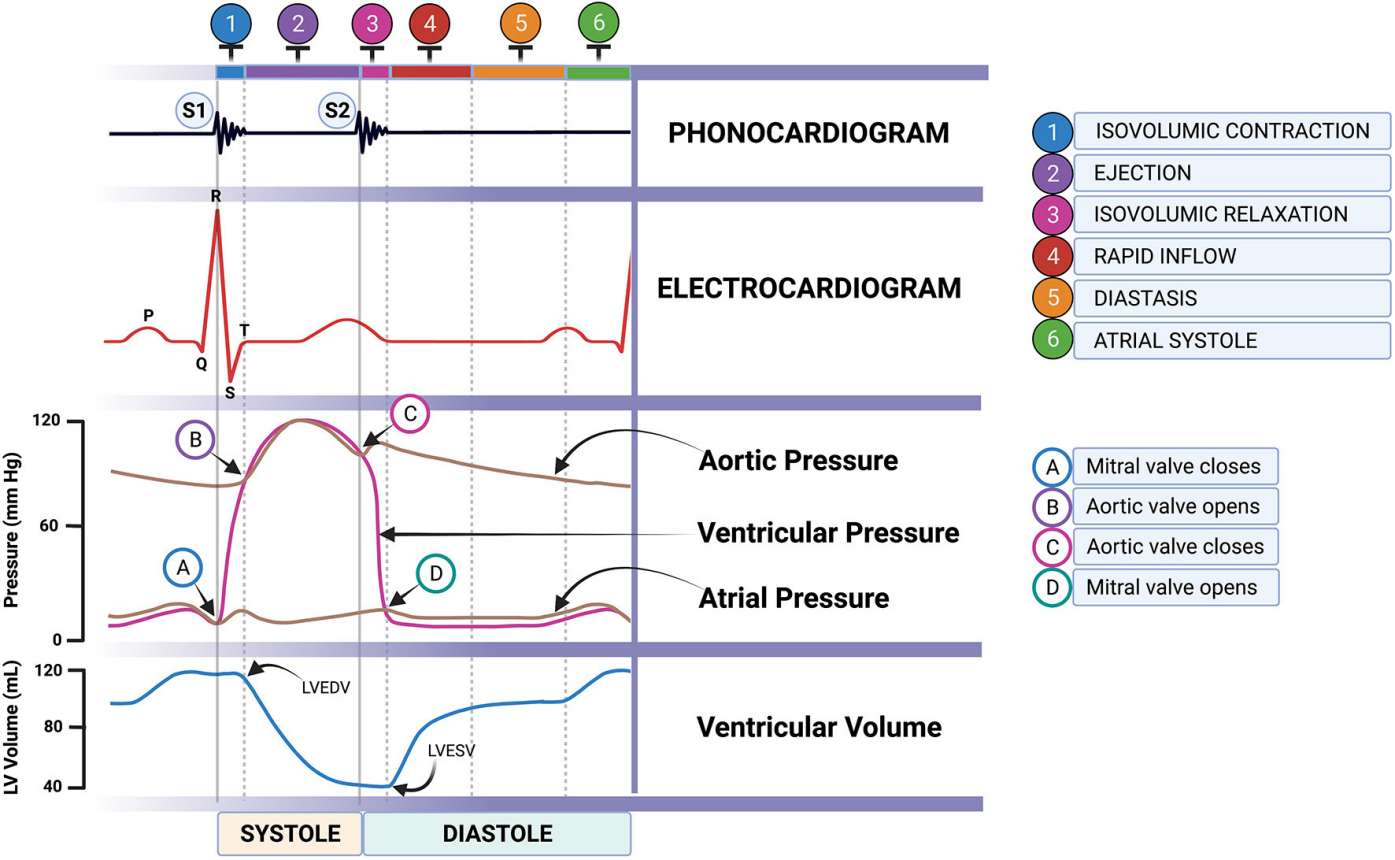
Cardiac cycle through the Wiggers diagram showing the simultaneous ECG and PCG recordings (artist: Bryan James P. Bulatao). PCG, phonocardiography.

Accessibility to an ultrasound machine, cost, and technical expertise are very challenging limitations in disease diagnosis. Hence, it is necessary to find a supportive diagnostic tool to describe cardiac function and associated pathologic changes. The simultaneous use of electrocardiography (ECG) and phonocardiography (PCG) can determine CTIs, providing information on the current state of the heart. These were proved as a useful tool in detecting LV dysfunction in humans including fetal heart function (26–28). In animals, it can be used to evaluate both systolic and diastolic functions (29) and allow early detection of acute myocardial ischemia (30). Recently, the digital stethoscope featuring ECG recording is commercially available, accessible, and affordable (Eko Duo ECG + Digital Stethoscope, Eko Devices Inc., Oakland, CA, USA). A similar device employing a digital stethoscope was used to determine heart murmurs and detect thrombosis in humans (31, 32). The evidence of the usefulness of this device in the early detection of reduced cardiac function can be beneficial to MMVD patients. According to the Wiggers diagram (Figure 1), the SPI can be alternatively measured by the simultaneous use of ECG and PCG. It can be calculated from QS1 duration divided by S1S2 duration (QS1/S1S2). Moreover, MPI may also be computed from three different equations [(QS1 + S2)/S1S2; (RS1 + S2)/S1S2; or RS1 + (QS2 – QT)/S1S2] mimicking the Tei index from echocardiography as we proposed. Whether these SPI and MPI from PCG and ECG correlate and reflect the cardiac function parameters, as measured by echocardiography, remains to be proven and elucidated in MMVD dogs.

The disease progression in MMVD causes changes in the electrical, mechanical, and hemodynamic properties of the heart affecting different cardiac timings and intervals. These changes can be valuable to describe and distinguish various stages of the disease. Therefore, we hypothesized that CTIs (i.e., SPI and MPI) derived simultaneously from ECG and PCG have potential support in the assessment of cardiac function in MMVD dogs.

Taken together, this study aimed to 1) establish SPI and MPI, using simultaneous recordings of ECG and PCG from the EkoDUO+ECG device, in dogs at different stages of MMVD; 2) to compare and correlate these derived CTIs with standard transthoracic echocardiography to illustrate various cardiac functions; and 3) lastly, to characterize various stages of MMVD and associate them with the asymptomatic and symptomatic patients using SPI and MPI. As a novel tool, these surrogate markers may benefit in detecting or screening a reduction of cardiac function in asymptomatic or symptomatic MMVD dogs with lower cost and are easily accessible for practitioners in the future.

## Materials and Methods

### Animals

The study engaged 52 client-owned MMVD dogs (26 female and 26 male) that visited the Cardiology Clinic at the Small Animal Teaching Hospital, Faculty of Veterinary Science, Chulalongkorn University. The handling and restraint of conscious dogs were in strict compliance with the approved institutional protocol (Institutional Animal Care and Use Committee 1931013, Faculty of Veterinary Science, Chulalongkorn University). In addition, written owner consent was secured for MMVD dogs prior to their inclusion in this study. All dogs were recruited from March 2020 to March 2021. These dogs have undergone physical and clinical examination to determine the presence of concurrent systemic and other valvular diseases. They were diagnosed and classified based on ACVIM guidelines (12) using thoracic radiography and standard trans-thoracic echocardiography. Dogs with concomitant systemic disease, congenital heart disease, and supraventricular and ventricular arrhythmias were excluded from this study. The dogs included in this study were actual clinical patients that were recruited by clinicians for MMVD diagnosis by an experienced sonologist. The subsequent CTI parameters were measured separately by the researcher, minimizing perceived bias. A separate researcher collected and grouped the data accordingly to the ACVIM guidelines (12). Briefly, dogs in B1 (*n* = 14) had murmur with no identification of left atrial (LA) and LV enlargement by thoracic radiography and echocardiography. B2 dogs (*n* = 14) were detected to have LA and LV enlargement using echocardiography (i.e., LA/aorta (Ao) ≥1.6 and LV internal diameter during diastole, normalized (LVIDDN) ≥1.7). Groups B1 and B2 had no clinical signs of CHF and were further classified as asymptomatic (*n* = 28). Groups C (*n* = 18) and D (*n* = 6) were dogs with clinical signs of CHF and present with left heart remodeling as confirmed by echocardiography. Dogs in group D were resistant to standard treatment from stage C. Groups C and D dogs were further assigned to the symptomatic group (*n* = 24) based on the presence of clinical signs of CHF (i.e., cough and dyspnea) and/or abnormalities in thoracic radiography (i.e., evidence of pulmonary edema). As recommended by the ACVIM guidelines (12), dogs in stages C and D were prescribed cardiovascular drugs such as furosemide, pimobendan, ACE inhibitor, aminophylline, spironolactone, and sildenafil depending on the severity of clinical signs. All dogs were clinically stable during the study.

### Recording of the Cardiac Time Intervals Using Eko Duo ECG + Digital Stethoscope

**Figure 2:**
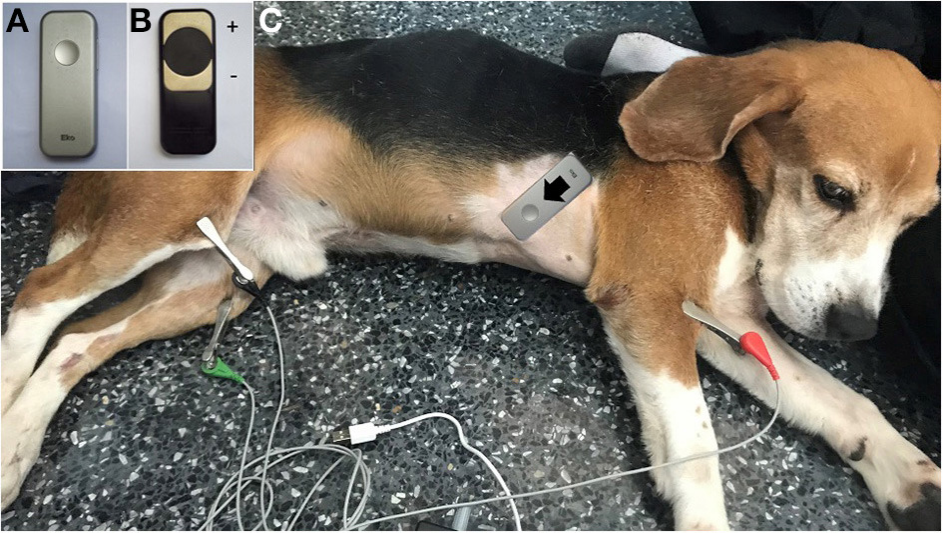
The Eko® DUO ECG+Stethoscope device in the top view **(A)** and bottom view **(B)** is labeled with positive and negative electrodes. The placement of the device in a dog at the right skin thorax at the direction from base to the apex of the heart **(C)**.

**Figure 3:**
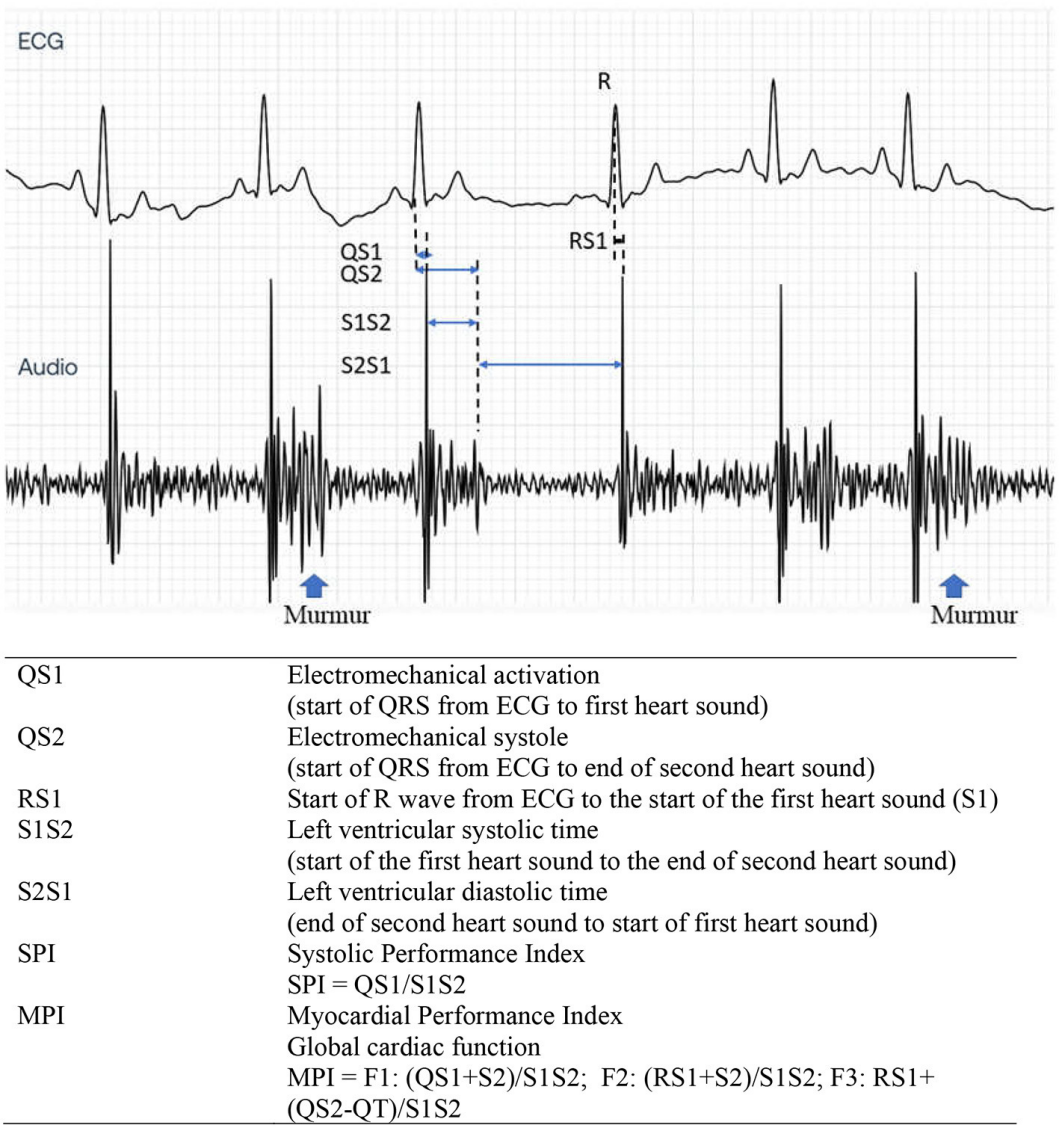
Representative tracing of ECG (top) and PCG (bottom) from the Eko analyzing software. Cardiac time interval recording and its description from a dog with stage C MMVD with heart murmurs. PCG, phonocardiography; MMVD, myxomatous mitral valve disease.

Various CTIs representing systolic and diastolic functions were measured simultaneously both using commercial devices utilizing ECG and PCG (Eko Duo ECG + Digital Stethoscope., Oakland, CA, USA) (Figure 2A) and the standard transthoracic echocardiography. The digital stethoscope part of this device has a frequency range of 20 Hz−2 kHz with a sample rate of 4,000 Hz. The ECG part utilized a single lead with two stainless steel electrodes (Figure 2B). The 500-Hz sampling rates were recorded by the device. The commercial device was placed on the right skin thorax of the animal while on left lateral recumbency (Figure 2C). The positive electrode was placed toward the apical portion, while the negative electrode was placed on the base of the heart, providing a base to apex vector ECG strip with simultaneous heart sound recordings. A graphical representation of the recorded ECG and PCG is shown in Figure 3. CTIs were recorded synchronously at the same beat recorded from the echocardiographic machine. These data were digitally recorded, sent, and stored wirelessly *via* Bluetooth(R) technology into the mobile phone using an application (Eko Devices, Inc. USA). This application utilized a digital caliper to measure various parameters, and values were reported in milliseconds (ms).

### Cardiac Time Interval Parameter Definitions and Measurement

In this study, we measured various CTI parameters representing electromechanical activation (QS1), electromechanical systole (QS2), LV systolic time (S1S2), LV diastolic time (S2S1), SPI, and MPI. These parameters signify systolic (QS1, QS2, SPI, and S1S2), diastolic (S2S1), and global cardiac (MPI) functions. These were measured using five subsequent simultaneous ECG and PCG recordings, and values were reported as time intervals (ms).

The measurement from the start of QRS in the ECG until the first (S1) and second (S2) heart sounds represented QS1 and QS2, respectively. Meanwhile, the distance from S1 until the end of S2 is characterized as the S1S2 parameter. The SPI was computed based on QS1/S1S2. In addition, the distance from the end of S2 until the next start of S1 (S2S1) indicated the diastolic time interval. In this study, we have proposed three new formulas for MPI as shown in Figure 3. The MPI was derived from existing CTI parameters using the equations (QS1 + S2)/S1S2; (RS1 + S2)/S1S2; or RS1 + (QS2 – QT)/S1S2 and designated as F1, F2, and F3, respectively.

### Echocardiography

**Figure 4:**
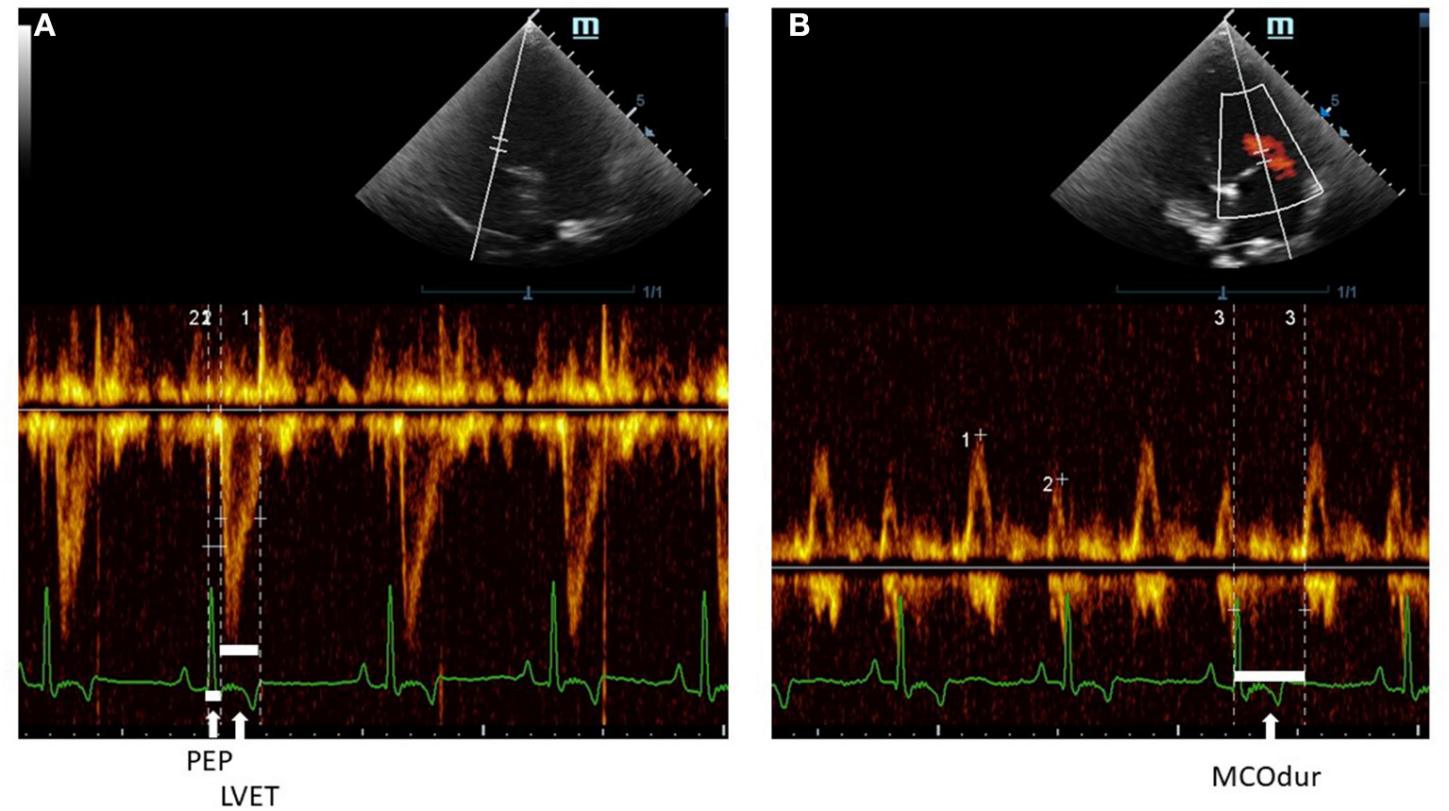
A Pulsed-wave Doppler echocardiographic measurement of **(A)** PEP and LVET and **(B)** MCOdur through the left apical view in a dog with MMVD. The Tei index was computed from (MCOdur – LVET)/LVET. PEP, pre-ejection period; LVET, left ventricular ejection time; MCOdur, mitral valve close to open duration; MMVD, myxomatous mitral valve disease.

Echocardiography was performed by one experienced echocardiographer to avoid inter-observer variation. To minimize intra-observer variation, an average of five consecutive beats during regular rhythm was reported in all parameters. The M9 Mindray® ultrasound machine (Shenzhen, China) was used for recording echocardiograms. The phased array ultrasound probe (Mindray^®^ P7-3s) was placed on the right and left skin thoraxes of the animal in lateral recumbency at the level of the third to fifth intercostal spaces. The animals were minimally restrained while placed on an ultrasound examination table. The standard ECG leads from the machine were clipped on the lower limbs. A right parasternal long-axis view was done to examine the overview of the heart chamber and the MV structure in B-mode. Abnormalities of the MV leaflets were confirmed in B-mode including thickening, prolapse, or both. In addition, the MR was confirmed by the color Doppler flow mode. For the right parasternal short-axis view, M-mode was performed to evaluate the cardiac dimensions and obtain the following parameters: Left atrium (LA), Aorta (Ao), LA/AO ratio, interventricular septum (IVS) during systole (IVSs) and diastole (IVSd), LV internal dimension (LVID) during systole (LVIDs) and diastole (LVIDd), and the LV posterior free wall (LVPW) during systole (LVPWs) and diastole (LVPWd). The percentage of fractional shortening (FS) was calculated based on the equation (LVIDd – LVIDs)/LVIDd × 100). All the M-mode echocardiographic values were normalized and calculated according to the allometric scaling of Cornell et al., (33). The ejection fraction (EF) was measured and calculated based on modified Simpson's and Teicholz methods at the left apical chamber view and in M-mode of short-axis view at the papillary muscle level, respectively. A pulsed-wave or continuous Doppler echocardiography was performed on the left apical four- or five-chamber views at the levels of trans-mitral inflow and LV outflow tracts (LVOTs) (34) to obtain CTI parameters. PEP, LVET, and MCO were measured from the LVOT flow profiles (Figure 4) together with heart rate (HR) from the ECG leads of the echocardiographic equipment. The MCO, mitral E/A, and IVRT were measured from the standard mitral inflow and LV outflow profiles. Global cardiac functions (Tei index) were computed from the equation [(MCO – LVET)/LVET]. All CTI parameters, from the Eko Duo ECG + Digital Stethoscope device and transthoracic echocardiography, were synchronously recorded. All CTIs were measured and reported as time intervals (ms) based on the average of five subsequent beats.

### Statistical Analysis

The values were reported as means (±SEM) to describe various MMVD stages based on ACVIM guidelines (B1, B2, C, and D) and the presence of clinical signs (asymptomatic and symptomatic). The data were analyzed using commercially available statistical software. The values were evaluated for normality using the Kolmogorov–Smirnov test. The description of dogs in the study was evaluated using ANOVA and post-hoc Duncan's test. On the other hand, the comparison between various CTI parameters of standard echocardiography and the device was analyzed using unpaired T, Mann–Whitney. The agreement between CTIs from the Eko Duo ECG + Digital Stethoscope device and echocardiography was done using the Bland–Altman test. The degree of relationship between these parameters was further investigated using Pearson's correlation test. The CTIs at different stages of MMVD were compared using ANOVA and post-hoc Duncan's tests. The different MPI formulas were evaluated for their area under the curve (AUC) using the receiver operating characteristic (ROC) test. All values were reported at a 95% confidence interval.

## Results

### Clinical Characteristics of Dogs

A total of 52 MMVD dogs, confirmed by standard echocardiography, were used in this study to investigate the use of CTIs in describing the different stages of MMVD. The animals were grouped based on the ACVIM guidelines (12). Table 1 demonstrates the general characteristics of dogs in the current study based on their sex, age, weight, breeds, and HR. In general, these parameters are equally distributed. There were no differences in the mean sex, age, and weight among MMVD groups. Twenty-six male and female MMVD dogs were included in this study. A lower number of dogs in stage D were seen as compared with other MMVD stages, while both stages B1 and C have a similar number of populations (*n* = 14). The mean age of all dogs was 12.07 ± 0.41 years, with 12.25 ± 0.59 years old in the asymptomatic group and 11.86 ± 0.58 years old in the symptomatic group. The mean weight of dogs (5.36 ± 0.35 kg) was not significantly different (*p* = 0.24) between the asymptomatic (5.73 ± 0.50 kg) and symptomatic (4.94 ± 0.46 kg) groups. The mean HR was at 146.79 ± 5.21 bpm and was increased by 25%−28% in stages B2, C, and D as compared with stage B1 but were not statistically different (*p* = 0.06) among groups. This study utilized small breed dogs, and the most common breeds include Chihuahua (13), Shih Tzu (12), Poodle (10), and Pomeranian (8).

**Table 1:** Description of dogs in different stages of MMVD in this study.

		**Asymptomatic** **(***n*** = 26)**		**Symptomatic** **(***n*** = 26)**		
		**B1**	**B2**	**C**	**D**	***p*** **-Value**
*n*		14	14	18	6	
Sex		M (8), F (6)	M (6), F (8)	M (10), F (8)	M (2), F (4)	0.05
Age (years)		13.07 ± 0.68	11.43 ± 0.93	11.68 ± 0.74	12.42 ± 0.82	0.46
Weight (kg)		6.52 ± 0.82	4.94 ± 0.53	4.85 ± 0.54	5.19 ± 0.94	0.24
HR (bpm)		123.57 ± 6.03	154.71 ± 6.67	154.83 ± 10.80	158.33 ± 19.23	0.06
Breed	Chihuahua	2	4	5	2	
	Mini Pinscher	1			1	
	Mixed	3			1	
	Pekingese			1		
	Pomeranian	1	3	4		
	Poodle	2	4	3	1	
	Schnauzer		1			
	Shih Tzu	4	2	5	1	
	Yorkshire Terrier	1				

*Data are expressed as mean ± SEM*.

*MMVD, myxomatous mitral valve disease*.

The echocardiographic profiles are listed in Table 2 to describe different MMVD stages. Both stages B1 and B2 were found to have significantly lower LA size than stages C (*p* = 0.02, *p* = 0.04) and D (*p* ≤ 0.0001, *p* ≤ 0.0001), respectively. Meanwhile, the Ao size was only seen significantly higher in stage B1 as compared with stage C (*p* = 0.03). The LA/Ao was increased significantly in stages C and D (*p* = 0.003 and *p* < 0.0001, respectively) when compared with stage B1, while stage B2 was also seen significantly lower compared with stage D (*p* = 0.002). These results indicate the progression of the enlarged LA in the advanced HF. Likewise, the LVIDDN values were observed to have an increasing trend as the MMVD stage progressed, reflecting LV volume overload. Stage B1 has the lowest LVIDDN value when compared with stages C (*p* = 0.001) and D (*p* = 0.006). There was an increasing trend observed as the MMVD stage progressed in various echocardiographic parameters including left ventricular internal diameter during systole, normalized (LVIDSN), EF (Teicholz and Simpson), FS, stroke volume (SV), and PEP/LVET. In contrast, there was a decreasing trend seen in PEP in higher stages of MMVD. However, these observations were not statistically significant.

**Table 2:** Echocardiographic parameters describing various stages of MMVD.

		**Asymptomatic**		**Symptomatic**		
	**Echo parameters**	**B1** **(***n*** = 14)**	**B2** **(***n*** = 14)**	**C** **(***n*** = 18)**	**D** **(***n*** = 6)**	
		**Mean ± SEM**	**Mean ± SEM**	**Mean ± SEM**	**Mean ± SEM**	***p*** **-value**
Dimensions	LA (cm)	1.71 ± 0.07^a^, ^b^	1.74 ± 0.09^a^, ^b^	2.15 ± 0.12^a^	2.77 ± 0.51	<0.0001
	Ao (cm)	1.23 ± 0.06^b^	1.05 ± 0.06	1.02 ± 0.04	1.10 ± 0.07	0.04
	LA/Ao	1.33 ± 0.04^a^, ^b^	1.69 ± 0.08^a^	2.14 ± 0.15	2.63 ± 0.37	<0.0001
	LVIDDN (cm)	1.30 ± 0.05^a^, ^b^	1.54 ± 0.10	1.82 ± 0.11	1.91 ± 0.15	0.0006
	LVIDSN (cm)	0.66 ± 0.03	0.72 ± 0.06	0.83 ± 0.08	0.82 ± 0.11	0.21
Cardiac function	EF Simpson (%)	55.32 ± 13.00	59.94 ± 4.98	69.10 ± 6.69	71.56 ± 2.11	0.49
	EF Teicholz (%)	79.00 ± 2.46	80.78 ± 2.63	85.53 ± 2.30	87.56 ± 2.63	0.12
	FS (%)	45.96 ± 2.45	49.36 ± 3.13	53.56 ± 3.30	57.43 ± 3.99	0.16
	SV (ml)	13.28 ± 2.33^a^	15.37 ± 2.24^a^	24.13 ± 3.90	36.07 ± 5.98	0.002
Systolic time interval	PEP (ms)	47.09 ± 2.92	46.07 ± 2.59	41.73 ± 2.23	36.12 ± 3.57	0.10
	LVET (ms)	167.60 ± 9.66^a^, ^b^	163.50 ± 18.73	126.00 ± 7.77	111.80 ± 9.04	0.01
	PEP/LVET	0.29 ± 0.02	0.33 ± 0.04	0.35 ± 0.03	0.33 ± 0.04	0.50
Diastolic time interval	MCOdur (ms)	219.90 ± 10.15^b^	209.70 ± 9.03	183.80 ± 6.82	184.40 ± 15.27	0.02
	Mitral E/A	0.87 ± 0.07^a^	0.90 ± 0.07^a^	1.2 ± 0.14^a^	2.1 ± 0.57	0.0007
	IVRT (ms)	69.35 ± 3.45	62.42 ± 5.94	53.36 ± 6.53	47.60 ± 3.46	0.10
Myocardial performance index	Tei	0.39 ± 0.05^a^	0.51 ± 0.10	0.62 ± 0.10	0.92 ± 0.25	0.048

*Data are expressed as mean ± SEM. The significant difference was assessed by one-way ANOVA and post-hoc Tukey's at p ≤ 0.05*.

*MMVD, myxomatous mitral valve disease; HR, heart rate; LA/Ao, left atrium/aorta; LVIDDN, left ventricular internal diameter during diastole, normalized; LVIDSN, left ventricular internal diameter during systole, normalized; EF, ejection fraction; FS, fractional shortening; SV, stroke volume; PEP, pre-ejection period; LVET, left ventricular ejection time; MCOdur, mitral valve close to open duration; Tei, myocardial performance index using standard echocardiography; IVRT, isovolumic relaxation time*.

a *Significant difference at p ≤ 0.05 compared with stage D*.

b *Significant difference at p ≤ 0.05 compared with stage C*.

The LVET values were seen to decrease in advance stages of MMVD, while stage B1 had the highest value as compared with stages C (*p* = 0.02) and D (*p* = 0.01). A similar decreasing trend was seen in MCOdur, with stage B1 significantly higher than stage C (*p* = 0.02). The mitral E/A was found significantly lower in stages B1 (*p* = 0.0007), B2 (*p* = 0.001), and C (*p* = 0.02) when compared with stage D. Meanwhile, IVRT did not differ across groups (*p* = 0.10). As a measure of MPI, the Tei index from echo was seen to increase in higher stages of MMVD, with stage B1 having the lowest value as compared with stage D (*p* = 0.04).

### Establishment of Cardiac Time Intervals From the Eko Duo ECG + Digital Stethoscope in Different Stages of Myxomatous Mitral Valve Disease Dogs

To establish several CTIs in different MMVD stages, we measured simultaneous recordings of ECG and PCG as shown in Table 3. The QS1 values were highest in stage D as compared with stages B1 (*p* = 0.04), B2 (*p* = 0.02), and C (*p* = 0.02), indicating a delay in electromechanical activation time. Similarly, stages B2 (*p* = 0.02) and C (*p* = 0.001) have lower QS2 (electromechanical systole) values than stage D. In S1S2 (an approximation of ejection time), stage D was higher compared with B2 (*p* = 0.006) and C (*p* = 0.0004), while stage B1 was higher than stage C (*p* = 0.01). These CTIs (i.e., QS1, QS2, and S1S2) were considered systolic time interval parameters, and all tend to increase at the end stage of the disease (D), suggesting a slow electromechanical coupling of these hearts. Meanwhile, diastolic time intervals (S2S1) and RR intervals were no different among MMVD groups.

**Table 3:** CTI parameters were measured using Eko^®^ DUO ECG+Stethoscope device in various stages of MMVD.

		**Asymptomatic**		**Symptomatic**		
**CTIs**		**B1** **(***n*** = 14)**	**B2** **(***n*** = 14)**	**C** **(***n*** = 18)**	**D** **(***n*** = 6)**	
		**Mean ± SEM**	**Mean ± SEM**	**Mean ± SEM**	**Mean ± SEM**	***p*** **-value**
QS1 (ms)		65.21 ± 3.55^a^	63.14 ± 3.79^a^	61.83 ± 2.71^a^	96.83 ± 25.80	0.02
QS2 (ms)		257.10 ± 7.72	246.40 ± 9.09^a^	230.00 ± 6.40^a^	301.50 ± 32.56	0.002
S1S2 (ms)		217.80 ± 9.90^b^	189.40 ± 8.33^a^	176.33 ± 6.85^a^	249.67 ± 23.16	0.0002
S2S1 (ms)		287.90 ± 22.02	294.00 ± 17.96	278.00 ± 14.47	268.97 ± 27.04	0.87
RR (ms)		467.20 ± 26.40	461.10 ± 22.39	451.17 ± 19.57	408.50 ± 48.25	0.61
SPI						
	QS1/S1S2	0.30 ± 0.02	0.34 ± 0.02	0.36 ± 0.02	0.37 ± 0.06	0.25
MPI						
	F1: (QS1 + S2)/S1S2	0.57 ± 0.02^b^	0.69 ± 0.05	0.75 ± 0.04	0.67 ± 0.05	0.006
	F2: (RS1 + S2)/S1S2	0.52 ± 0.02^b^	0.63 ± 0.04	0.67 ± 0.03	0.51 ± 0.05	0.009
	F3: RS1 + (QS2 – QT)/S1S2	0.55 ± 0.04	0.61 ± 0.05	0.54 ± 0.03	0.63 ± 0.06	0.42

*Data are expressed as means ± SEM. The significant difference was assessed by one-way ANOVA at p ≤ 0.05 and post-hoc Duncan's test*.

*CTI, cardiac time interval; MMVD, myxomatous mitral valve disease; SPI, systolic performance index; MPI, myocardial performance index*.

a *Significant difference at p ≤ 0.05 compared with stage D*.

b *Significant difference at p ≤ 0.05 compared with stage C*.

**Figure 5:**
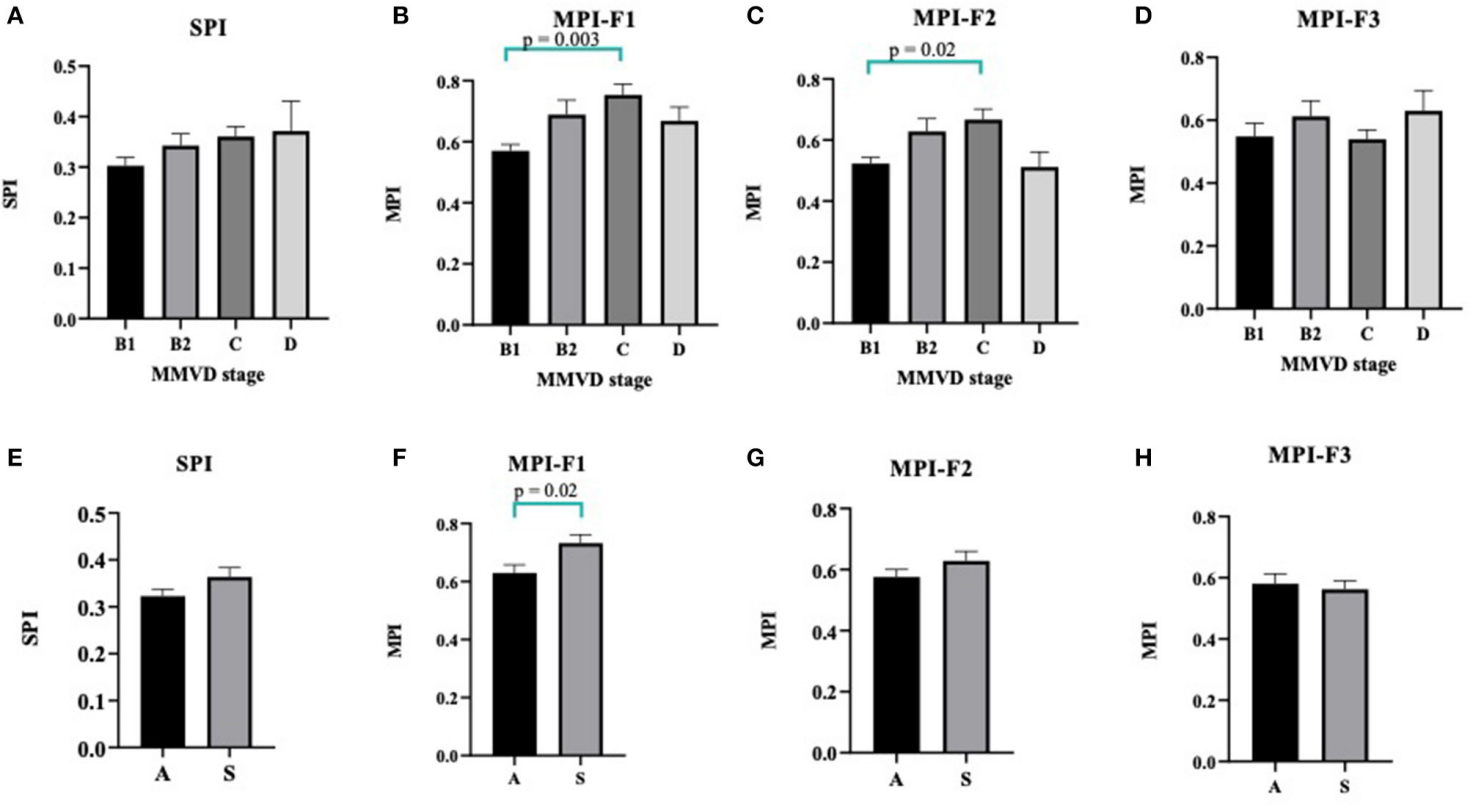
Column bars represented mean CTI values of MMVD stages of SPI **(A)**, MPI-F1 **(B)**, MPI-F2 **(C)**, and MPI-F3 **(D)**. Data were expressed as mean ± SEM and were compared using ANOVA and post-hoc Tukey's test at *p* < 0.05. For asymptomatic [A] vs. symptomatic [S] MMVD, CTI values of SPI **(E)**, MPI-F1 **(F)**, MPI-F2 **(G)**, and MPI-F3 **(H)**. Data were expressed as mean ± SEM and compared using unpaired *t*-test at *p* < 0.05. CTI, cardiac time interval; MMVD, myxomatous mitral valve disease; SPI, systolic performance index; MPI, myocardial performance index.

The SPI values as calculated from QS1/S1S2 tend to be increased in reference to higher stages of MMVD, but this trend was not statistically significant (Figure 5A). As we proposed above, three different equations to estimate the MPI in MMVD groups were shown in Figure 3 and labeled as F1, F2, and F3. The mean values of MPI-F1 were increased significantly in stage C (0.75 ± 0.04) when compared with stage B1 (0.57 ± 0.02) (*p* = 0.003) (Figure 5B). This result was similar to MPI-F2, where stage B1 (0.53 ± 0.02) was significantly lower than stage C (0.67 ± 0.03) (*p* = 0.02) (Figure 5C). This trend however went down at stage D. There were no differences seen in MPI-F3 in the different stages of MMVD (Figure 5D).

### Differentiation of Asymptomatic or Symptomatic Myxomatous Mitral Valve Disease Dogs Using Cardiac Time Interval From the Device

To further compare whether the SPI and MPI derived from CTIs are different between asymptomatic and symptomatic MMVD dogs, we compared these data as shown in Figures 5E–H. Interestingly, the MPI-F1 significantly increased in the symptomatic (S) (0.73 ± 0.03) as compared with asymptomatic (A) (0.63 ± 0.03) dogs (*p* = 0.02) and can be used to characterize between the two groups (Figure 5F), whereas SPI, MPI-F2, and F3 could not be differentiated in these dogs (Figures 5E,G,H, respectively).

### Comparison Between the Echocardiographic Cardiac Time Interval and the Cardiac Time Interval Derived From Eko Duo ECG + Digital Stethoscope

**Figure 6:**
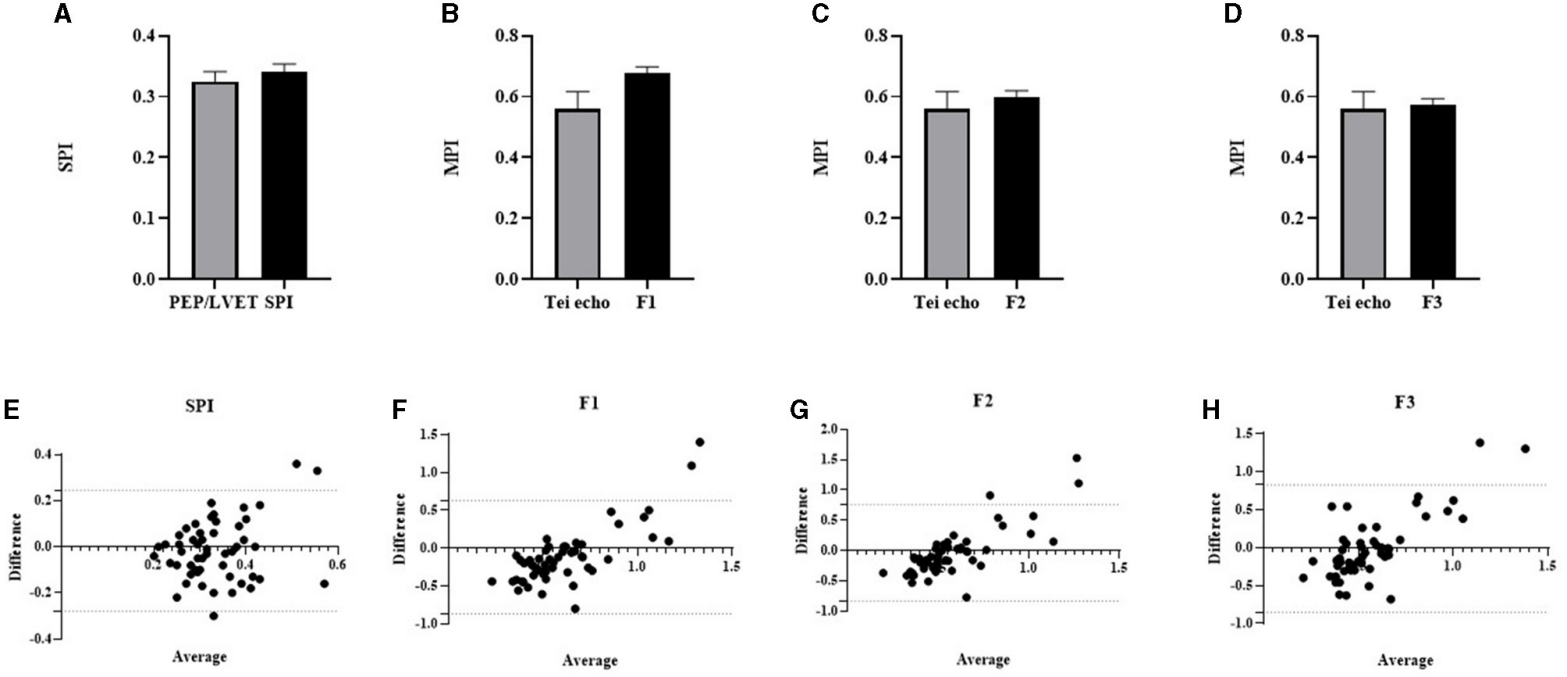
Column bar graph of mean CTI parameters from Eko^®^ DUO ECG+Stethoscope device and standard trans-thoracic echocardiography were compared: PEP/LVET vs. SPI **(A)**; Tei index from echo vs. MPI-F1 **(B)**; Tei index from echo vs. MPI-F2 **(C)**; Tei index from echo vs. MPI-F3 **(D)**. Data were expressed as mean ± SEM and compared using unpaired *t*-test at *p* ≤ 0.05. The Bland–Altman test for the two different methods was used to compare PEP/LVET and SPI **(E)**, Tei echo and MPI-F1 **(F)**, Tei echo and MPI-F2 **(G)**, and MPI-F3 **(H)**. The horizontal dash lines indicate 95% CI. CTI, cardiac time interval; PEP, pre-ejection period; SPI, systolic performance index; MPI, myocardial performance index; LVET, left ventricular ejection time.

To further investigate whether CTIs from both standard trans-thoracic echocardiography and the device differ, we compared these values in Figure 6. There were no significant differences in the mean values observed between these parameters from standard echocardiography and the device. A systolic function parameter, the SPI was compared using QS1/S1S2 (0.34 ± 0.01) from the device and PEP/LVET (0.33 ± 0.02) from standard echocardiography (Figure 6A). As a measure of global cardiac function, the MPI was derived using the three proposed formulas. The mean average calculated from all proposed formulas did not significantly differ (F1: 0.68 ± 0.02; F2: 0.60 ± 0.02; F3: 0.57 ± 0.02) from the standard Tei index (0.56 ± 0.06) of trans-thoracic echocardiography (Figures 6B–D). The agreements between echocardiographic SPI (PEP/LVET) and MPI (Tei index) and the device SPI (QS1/S1S2) and MPI (F1, F2, and F3) were assessed using the Bland–Altman plot as shown in Figures 6E–H. The SPI had a bias of −0.02 (−0.28 to 0.25, 95% CI). The observed bias of MPI was −0.12 (−0.87–0.63, 95% CI), −0.04 (−0.84–0.76, 95% CI), and −0.01 (−0.85–0.83, 95% CI) for F1, F2, and F3, respectively when compared with the Tei index. In general, data from both methods agreed and fell within 95% CI.

### Relationship Between the Cardiac Time Interval Derived From Eko Duo ECG + Digital Stethoscope and Cardiac Time Interval From Echocardiography

We continued to determine the relationships of SPI and MPI with other standard echocardiographic parameters (Table 4). The HR was seen moderately correlated with both MPI-F1 and F2 (*p* = 0.01, *r* = 0.35; and *p* = 0.003, *r* = 0.41, respectively). Because of HR dependency, MPI-F1 and F2 can be corrected using the slope of regression line for HR vs. F1 (i.e., F1 = 0.001411 ^*^ HR + 0.4698) and F2 (i.e., F2 = 0.001547 ^*^ HR + 0.3729). There was no significant correlation observed in the cardiac dimension and cardiac function of standard echocardiography with SPI and MPI from the device. The PEP, a measurement of systolic time interval from echocardiography, was seen as negatively correlated with MPI-F2 (*p* = 0.03, *r* = −0.30). The LVET was also seen as negatively correlated with MPI-F1 (*p* = 0.02, *r* = −0.33), MPI-F2 (*p* = 0.01, *r* = −0.36), and MPI-F3 (*p* = 0.04, *r* = −0.29). Meanwhile, PEP/LVET, MCOdur, mitral E/A, and IVRT did not show any significant relationship with SPI and MPI. Interestingly, the SPI and MPI-F1 were both observed to be significantly correlated with the Tei index (*r* = 0.31, *p* = 0.03; and *r* = 0.37, *p* = 0.01, respectively). Therefore, it may be suitable to use the MPI-F1 to determine the Tei index by applying the slope of regression line for the Tei index vs. MPI-F1 (Tei index = 0.9851 ^*^ F1 – 0.1068). Among the three MPIs, only MPI-F1 seems to be a good diagnostic parameter for global cardiac function.

**Table 4:** Relationship of CTI parameters between standards trans-thoracic echocardiography and simultaneous ECG and PCG recordings.

		**SPI**	**F1**	**F2**	**F3**
	HR	0.22, 0.12	0.35, 0.01^*^	0.41, 0.003^**^	0.13, 0.35
Dimension	LA/Ao	0.11, 0.45	0.16, 0.26	0.07, 0.64	0.09, 0.53
	LVIDDN (cm)	−0.03, 0.81	0.02, 0.90	0.01, 0.93	−0.07, 0.63
	LVIDSN (cm)	−0.14, 0.34	0.03, 0.81	0.06, 0.66	−0.13, 0.37
Cardiac function	EF Simpson (%)	−0.23, 0.36	−0.19, 0.46	−0.25, 0.32	−0.29, 0.25
	EF Teicholz (%)	−0.01, 0.92	−0.09, 0.53	−0.10, 0.49	0.02, 0.90
	FS (%)	0.12, 0.41	0.07, 0.62	−0.03, 0.84	0.15, 0.31
	SV (ml)	−0.08, 0.60	−0.09, 0.51	−0.17, 0.24	0.19, 0.17
Systolic time interval	PEP (ms)	−0.02, 0.90	−0.27, 0.05	−0.30, 0.03^*^	−0.05, 0.75
	LVET (ms)	−0.14, 0.33	−0.33, 0.02^*^	−0.36, 0.01^**^	−0.29, 0.04^*^
	PEP/LVET	0.16, 0.24	0.13, 0.34	0.10, 0.47	0.23, 0.11
Diastolic time interval	MCOdur (ms)	−0.05, 0.71	−0.24, 0.08	−0.27, 0.05	−0.05, 0.75
	E/A	0.09, 0.54	0.18, 0.21	0.13, 0.36	0.13, 0.38
	IVRT (ms)	−0.14, 0.92	−0.14, 0.33	−0.16, 0.27	0.01, 0.94
Global cardiac function	Tei	0.31, 0.03^*^	0.37, 0.01^**^	0.21, 0.13	0.08, 0.56

Data are presented as correlation coefficient with p-value (r, p-value), where ^*^, correlation is significant at p < 0.05 and ^**^, correlation is significant at p < 0.01 using Pearson's correlation test.

*HR, heart rate; LA/Ao, left atrium/aorta; LVIDDN, left ventricular internal diameter during diastole, normalized; LVIDSN, left ventricular internal diameter during systole, normalized; EF, ejection fraction; FS, fractional shortening; SV, stroke volume; PEP, pre-ejection period; LVET, left ventricular ejection time; MCOdur, mitral valve close to open duration; Tei, myocardial performance index using standard echocardiography; IVRT, isovolumic relaxation time; SPI, systolic performance index*.

*SPI (QS1/S1S2), F1 ((QS1+S2)/S1S2), F2 ((RS1+S2)/S1S2), and F3 ((QS2 – QT)/S1S2)*.

### Myocardial Performance Index-F1 and Tei Index in Differentiating Myxomatous Mitral Valve Disease Stages

**Figure 7:**
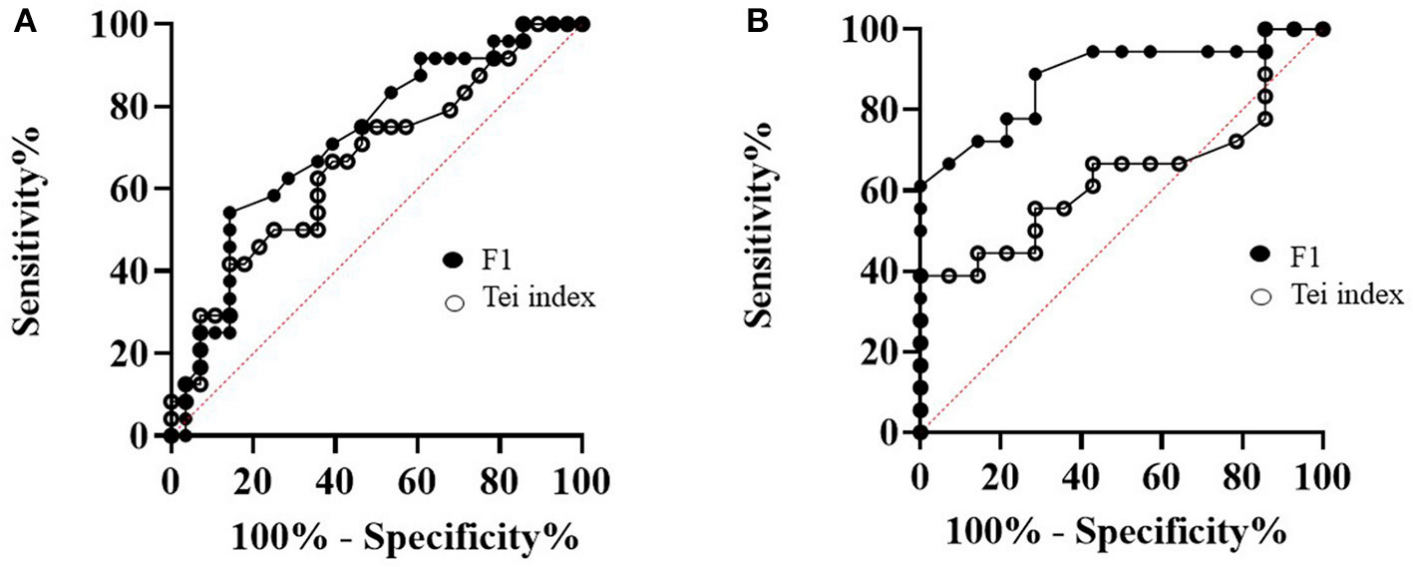
A comparison of the receiver operating characteristic (ROC) curves of MPI-F1 and Tei index between asymptomatic and symptomatic MMVD dogs **(A)** and B1 and C MMVD dogs **(B)**. MPI, myocardial performance index; MMVD, myxomatous mitral valve disease.

The ROC curves were constructed to identify whether MPI-F1 would help in the prediction of MMVD stages and compared with the Tei index from echocardiography and shown in Figure 7. We evaluated its sensitivity and specificity as tools for MMVD stage diagnosis. In Figure 7, MPI-F1 and the Tei index were evaluated based on their AUC of ROC at a 95% confidence interval. A comparison AUC between asymptomatic and symptomatic MMVD dogs is shown in Figure 7A. The AUC of MPI-F1 indicates moderate discriminatory ability (0.72, *p* = 0.007, 0.58–0.86 at 95% CI) and was higher than the Tei index (AUC = 0.66, *p* = 0.04, 0.52–0.81 at 95% CI). MPI-F1 had 71% sensitivity and 61% specificity, while the Tei index had 67% sensitivity and 61% specificity. Since the MPI-F1 was significantly higher in stage C when compared with stage B1, we also run the ROC curve of MPI-F1 and the Tei index between both groups (Figure 7B). MPI-F1 consistently recorded higher AUC (0.88, *p* = 0.0003, 0.76–1.0 at 95% CI) than the Tei index (0.64, *p* = 0.18, 0.44–0.83 at 95% CI). The MPI-F1 had high sensitivity (89%) and specificity (71%), while the Tei index demonstrated lower sensitivity (67%) and specificity (57%).

## Discussion

The current study established the CTI parameters, especially the SPI and MPI, measured by the novel commercially available device in MMVD dogs and compared with those parameters obtained from the standard echocardiography. Both cardiac function parameters (i.e., SPI and MPI) were comparable with the value measured by echocardiography. In addition, we proposed a suitable equation derived from CTIs as an estimation of MPI measurement. Our proposed MPI-F1 formula demonstrated a good prognostic value in differentiating asymptomatic and symptomatic MMVD dogs. The MPI-F1 was seen as a good supportive diagnostic tool when compared with the Tei index from standard trans-thoracic echocardiography in differentiating MMVD. Since CTIs were not fully elucidated and described in MMVD dogs, we believe that our data provided the first description of these parameters for clinical use and will be useful in the practice to measure cardiac global function where the echocardiography machine is not available.

Consistent with previous reports, we found that our recruited MMVD dogs in this study are small breeds and aged dogs (1–3). These dogs were classified based on ACVIM guidelines (4), in which LA/Ao ratio and LVIDDN from echocardiography were used to differentiate stage B1 from B2. In addition, the presence of clinical signs was used to separate asymptomatic and symptomatic dogs. We had similar numbers of asymptomatic and symptomatic aged-match dogs among all MMVD stages. However, there were a fewer number of dogs in stage D because of the difficulty in finding clinically stable stage D dogs with the permission of the owner to enroll in this study.

Pathophysiology of MMVD involves the degeneration of the MV structure that causes the regurgitation of blood flow resulting in volume overload of the LA and LV. Consequently, the cardiac dimension is increased and activates signaling pathways that promote cardiac remodeling (12). Previous studies highlighted the significance of these structural changes in the context of MMVD disease progression as seen in the vertebral heart score (VHS) (35) and vertebral LA size (VLAS) (36, 37) using thoracic radiography and the cardiac dimension changes in echocardiography (7, 12, 13). Eventually, these structural changes alter the systolic (12) and/or the diastolic (13, 14) functions. The reduction in cardiac function and cardiac output, then, triggers compensatory mechanisms including sympathetic activity that augmented the systolic function of the heart. Normally, the systolic function of the MMVD patients will be well-preserved by this mechanism until the end stage of HF. As the disease progressed, alterations in the structure and cardiac function may affect the various CTIs within the cardiac cycle.

As expected, we observed the disease progression as the LA, LA/Ao, and LVIDDN were increased in stages B1–D MMVD dogs. The increased measurements of echocardiographic parameters were seen to be related to cardiac mortality in dogs with MMVD (7, 38). Systolic function parameters (FS and EF) as assessed by echocardiography are within normal limits and tend to increase among the MMVD groups but were not different. This may be explained by the compensatory mechanism, or these dogs were prescribed with the positive inodilator (pimobendan) as recommended by the ACVIM guidelines. The previous report demonstrated that SPI and PEP/LVET are sensitive indicators of diminished LV performance and are useful for distinguishing normal from abnormal cardiac function in dogs (39). The SPI changes are expected with HF, becoming highlighted as myocardial failure develops. Therefore, these results implied that dogs in our study have well-conserved myocardial systolic performance. The mitral E/A values of stages B1, B2, and C were within the normal limit of 1.46–1.48 (34) when compared with higher values than normal in stage D dogs, indicating the presence of diastolic dysfunction. However, this was not consistently observed in IVRT values at different stages. The conserved systolic and diastolic functions may be influenced by the compensatory mechanism and the presence of medication. The Tei index in MMVD dogs was reported previously using a (human) modified New York Heart Association (NYHA) or International Small Animal Cardiac Health Council (ISACHC) classification system (17, 25). The Tei value from those published articles was ranging from 0.47 to 0.94 along with the increment class (I–IV) of heart disease. In our study, the Tei index of MMVD dogs according to stage definition by ACVIM has a similar trend with the previous reports (17, 25). The MPI or Tei index has been suggested as a reliable echocardiographic parameter for evaluating global cardiac function in both humans and small animals. In our study, the Tei index in stage D dogs has significantly increased even though all systolic parameters were within normal limits. This result suggests that the Tei index is a sensitive indicator in detecting a decline in cardiac function particularly the diastolic function component.

We established various CTIs parameters in MMVD dogs for the first time using the Eko Duo ECG + Digital Stethoscope device. There are several advantages of using this device such as affordability, being lightweight, and an easy-to-use analysis software. We found increased values in QS1, QS2, S1S2, and MPI formulas in the advanced stages of the disease. In humans, it was reported that QS1, QS2, and S1S2 increased in patients with HF and LV dysfunction (22, 26). However, there were no reported MPI values derived from ECG and PCG recordings reported in cardiac diseases including MMVD in dogs and humans. Changes in CTIs were also observed in valvular diseases in both humans (15, 16) and dogs (17, 18). These CTI changes reflect the systolic and diastolic dysfunctions associated with MMVD progression and remodeling (40–43). On the other hand, the non-significant differences in S2S1 and RR among MMVD stages may be influenced by several factors including the compensatory mechanisms of the heart, medication, or the absence of reported arrhythmias in the patients.

Since there were observed CTI changes in MMVD stages, we compared CTI parameters from standard echocardiography and simultaneous ECG and PCG recordings. The device's CTI parameters were comparable with the echocardiographic CTI parameters, indicating its potential prognostic use in MMVD dogs. This finding is coherent with another study in which the use of CTI parameters from acoustic cardiography was also interchangeable with standard echocardiographic values (24). The CTIs were also seen comparable with highly invasive pressure–volume measurements in myocardial ischemia models (30). Thus, CTI from the device is a possible supportive tool in describing cardiac function together with CTIs from standard echocardiography.

Systolic time intervals are useful in describing changes in cardiac function and differentiating phenotypes of cardiac-related diseases (22, 26, 44, 45). The SPI was reported to be strongly correlated with LVEF and an independent predictor of LV dysfunction in humans (26). In the same direction with the PEP/LVET, we did not find any differences in SPI (QS1/LVET) across MMVD stages, but there was a tendency to increase in the higher stage of MMVD. Therefore, the use of SPI from the device may not be sensitive enough to differentiate MMVD staging in dogs under recommended treatment. Rather, it would be useful to detect LV dysfunction in dilated cardiomyopathy (DCM) patients. The observed decreased MCOdur value in stage C dogs, when compared with stage B1 dogs, can possibly be influenced by the treatment, management, improvement of clinical signs, and/or current health state of the patient.

In humans, the development of MPI (Tei index) from non-invasive Doppler echocardiography (46) correlated well with invasive measurements of cardiac function. This tool was able to distinguish the presence of acute myocardial infarction (47), MR (48), LV dysfunction (49, 50), and CHF (51, 52). In dogs, higher MPI was observed in the presence of LV dysfunction (53), MR (17), and MMVD (25). In agreement with previous studies, we have observed higher MPIs in advanced stages of MMVD from both the standard trans-thoracic echocardiography and the device. We have proposed three different estimations of MPIs based on the measurement of cardiac timing of the Wiggers diagram. All three proposed MPIs from the device agreed with the measured Tei index and correlated well with it. In addition, all these MPIs also correlated well with LVET consistently with previous reports (17). Remarkably, this study was able to demonstrate that the proposed stages B1 and C have different MPI-F1 and MPI-F2 values, while only MPI-F1 can reliably distinguish asymptomatic from symptomatic MMVD dogs. The use of the Tei index was reported to have a good prognostic value in diagnosing MMVD in dogs (54). In comparison with the standard Tei index, MPI-F1 demonstrated good predictive accuracy in distinguishing asymptomatic from symptomatic dogs and considerably a supportive tool in differentiating these dogs. Therefore, MPI-F1 can be considered as a supportive tool in the diagnosis of MMVD patients.

In veterinary medicine, echocardiography has been a gold standard tool in the diagnosis and staging of MMVD dogs. We emphasized here that it is still necessary to use echocardiography as the definitive diagnostic tool for MMVD, since the degenerative MV leaflet and the MR can be visualized and confirmed by both 2D and Doppler echocardiography. However, there are several limitations including the need for a skilled sonographer and an experienced cardiologist to interpret images and measure parameters, the cost of the machine, and the lack of accessibility in the remote area. Normally, digital stethoscopes provide the PCG that allows an inexperienced veterinarian to visualize the abnormal heart sound. By coupling this with an ECG, the device provides more information regarding cardiac function and might be a useful tool for general practitioners. This will aid them in describing the cardiac function of MMVD patients especially in areas where a standard echocardiographic examination is limited or inaccessible, hence increasing diagnostic confidence before referral to a specialist.

### Limitations of the Study

This study has a limited number of dogs with stage D MMVD, which may not fully represent the CTI values in this group. Nevertheless, our data provided a stark difference in CTI values between the initial and advanced stages of the disease. The CTIs can be affected by various factors such as preload, myocardial contractility, HR, and afterload (39). However, a previous study (17) in MR dogs suggested that these factors have less influence when CTIs were measured in static conditions (e.g., no change in preload or afterload) as occurred in our study. In addition, HR and BW were found to have no correlation with the Tei index measured in MR dogs (17). In contrast, we found that HR is correlated with MPI-F1 and MPI-F2, potentially affecting the values in cases of severe arrhythmia. We have excluded MMVD dogs with supraventricular and ventricular arrhythmias in our study and hope to minimize these influences by the arrhythmia. However, it will be important to consider the presence of arrhythmias in the future use of CTIs for disease diagnosis. Dogs in various stages of MMVD received medications based on the ACVIM guidelines. These drugs can have various effects in MMVD dogs such as changes in the cardiac function to delay the progression of the disease and improve life quality and survival (55–58). These may potentially affect the various CTIs at different MMVD stages. Nevertheless, the CTIs reported in this study reflect the real clinical cases that veterinarians may encounter during their practice. Since the pathogenesis of heart disease is various and depends on the root cause, the CTIs reported in this study may be valid for MMVD dogs. Further study is required to investigate the potential uses of the device and our proposed MPI-F1 in other heart diseases such as the DCM. In addition, the usefulness of these CTI parameters compared with other indices (such as VLAS or VHS) to predict MMVD stages was not investigated in this study and can be considered in future research.

## Conclusion

Our study demonstrated that CTIs are measurable in MMVD dogs by using simultaneous recordings of ECG and PCG from a commercially available device. Various CTI values using this method in dogs were established in different stages of MMVD. We have successfully validated the prognostic value of our proposed MPI-F1 (i.e., QS1 + S2/S1S2) in MMVD dogs. The MPI-F1 was comparable and associated with the Tei index from standard trans-thoracic echocardiography. This estimation of the Tei index by MPI-F1 was different between asymptomatic and symptomatic dogs with good predictive accuracy, sensitivity, and specificity, thus making it a novel parameter that is accessible, accurate, cheap, and convenient to use that will aid in evaluating a change in the global cardiac function of MMVD patients.

## Data Availability Statement

The original contributions presented in the study are included in the article/supplementary material, further inquiries can be directed to the corresponding author/s.

## Ethics Statement

The animal study was reviewed and approved by Institutional Animal Care and Use Committee, Faculty of Veterinary Science, Chulalongkorn University, Bangkok, Thailand. Protocol no. 1931013. Written informed consent was obtained from the owners for the participation of their animals in this study.

## Author Contributions

KT: conceptualization, project administration, data collection and analysis, and manuscript writing. KG: data collection, data analysis, and manuscript writing. TL: data collection. All authors contributed to the article and approved the submitted version.

## Funding

This study was funded by the 90th Anniversary of Chulalongkorn University Scholarship (to KT and KG) and the 100th Anniversary Chulalongkorn University for Doctoral Scholarship (to KG).

## Conflict of Interest

The authors declare that the research was conducted in the absence of any commercial or financial relationships that could be construed as a potential conflict of interest.

## Publisher's Note

All claims expressed in this article are solely those of the authors and do not necessarily represent those of their affiliated organizations, or those of the publisher, the editors and the reviewers. Any product that may be evaluated in this article, or claim that may be made by its manufacturer, is not guaranteed or endorsed by the publisher.

## Abbreviations

ACVIM: American College of Veterinary Internal Medicine
CHF: congestive heart failure
CTI: cardiac time interval
DCM: dilated cardiomyopathy
ECG: electrocardiography
EF: ejection fraction
HR: heart rate
IVCT: isovolumic contraction time
IVRT: isovolumic relaxation time
IVSd: interventricular septum during diastole
IVSs: interventricular septum during systole
LV: left ventricle
LVET: left ventricular ejection time
LVIDd: left ventricular internal dimension during diastole
LVIDs: left ventricular internal dimension during systole
LVPWd: left ventricular posterior free wall during diastole
LVPWs: left ventricular posterior free wall during systole
MCO/MCO: durmitral valve close to open duration
MMVD: myxomatous mitral valve disease
MPI: myocardial performance index
MR: mitral regurgitation
PCG: phonocardiography
PEP: pre-ejection period
SPI: systolic performance index
VHS: vertebral heart size
VLAS: vertebral left atrial size.
